# Activating AhR alleviates cognitive deficits of Alzheimer's disease model mice by upregulating endogenous Aβ catabolic enzyme Neprilysin

**DOI:** 10.7150/thno.61601

**Published:** 2021-08-11

**Authors:** Cheng Qian, Chunjie Yang, Mengting Lu, Jiaxin Bao, Haiyan Shen, Bingquan Deng, Shensen Li, Wenwen Li, Mu Zhang, Changchun Cao

**Affiliations:** 1 Sir Run run Hospital, Nanjing Medical University, Nanjing, China.; 2 State Key Laboratory of Natural Medicines, China Pharmaceutical University, Nanjing, China.

**Keywords:** Neprilysin, Aryl hydrocarbon receptor, Anti-amyloidogenic therapy, Amyloid-β, Alzheimer's disease

## Abstract

**Rationale:** Neprilysin (NEP) is a major endogenous catabolic enzyme of amyloid β (Aβ). Previous studies have suggested that increasing NEP expression in animal models of Alzheimer's disease had an ameliorative effect. However, the underlying signaling pathway that regulates NEP expression remains unclear. The aryl hydrocarbon receptor (AhR) is a ligand-activated cytoplasmic receptor and transcription factor. Recent studies have shown that AhR plays essential roles in the central nervous system (CNS), but its physiological and pathological roles in regulating NEP are not entirely known.

**Methods:** Western blotting, immunofluorescence, quantitative RT-PCR and enzyme activity assay were used to verify the effects of AhR agonists on NEP in a cell model (N2a) and a mouse model (APP/PS1). Luciferase reporter assay and chromatin immunoprecipitation (ChIP) assay were conducted to investigate the roles of AhR in regulating NEP transcription. Object recognition test and the Morris water maze task were performed to assess the cognitive capacity of the mice.

**Results:** Activating AhR by the endogenous ligand L-Kynurenine (L-KN) or FICZ, or by the exogenous ligand diosmin or indole-3-carbinol (I3C) significantly increases NEP expression and enzyme activity in N2a cells and APP/PS1 mice. We also found that AhR is a direct transcription factor of NEP. Diosmin treatment effectively ameliorated the cognitive disorder and memory deficit of APP/PS1 transgenic mice. By knocking down AhR or using a small molecular inhibitor targeting AhR or NEP, we found that diosmin enhanced Aβ degradation through activated AhR and increased NEP expression.

**Conclusions:** These results indicate a novel pathway for regulating NEP expression in neurons and that AhR may be a potential therapeutic target for the treatment of Alzheimer's disease.

## Introduction

Abnormal accumulation of amyloid β (Aβ) in the brain plays a crucial role in the pathology of Alzheimer's disease (AD) 1. It has been reported that failure of clearance but not overproduction of Aβ is found in late-onset AD patients, which may relate to Aβ deposition and plaque formation 2. Therefore, enhancing Aβ clearance is a potential therapeutic strategy for AD. Aβ in the brain is cleared mainly through degradation by a family of amyloid degrading enzymes (ADEs), phagocytosis by microglia and astrocytes, and transport from the brain and proteolytic removal in the periphery 3. In recent years, a growing number of studies have found that enhancing the expression or activity of ADE effectively regulated Aβ levels and further ameliorated learning and memory deficiency of transgenic animal models 4.

Neprilysin (NEP), also known as neutral endopeptidase, is a plasma membrane zinc metallopeptidase. NEP is the major Aβ degrading enzyme both in the brain and in the periphery 5-7. The localization of NEP inversely correalates with Aβ pathology 8. Genetic NEP deficiency leads to defects in the degradation of both exogenous and endogenous Aβ, which results in abnormal accumulation of the toxic isoform Aβ42 in the brain 9, 10. The expression and activity of NEP decrease during aging and AD progression, which may contribute to AD development 11-14. In contrast, an increasing the expression and activity of NEP has been shown to reduce Aβ deposition and rescue cognitive deficiency in transgenic animal models 15-17. Thus, development of agents that upregulate brain NEP expression and activity may provide effective therapeutic results.

The aryl hydrocarbon receptor (AhR) is a ligand-activated cytoplasmic receptor and transcription factor 18. In addition to regulating detoxification, AhR plays essential roles in neuronal, immune, cardiovascular, hepatic, hematopoietic and reproductive systems 19. AhR is widely expressed in the central nervous system (CNS), but its physiological and pathological roles are not entirely known. Knock out of AhR in mice resulted in locomotor defects and alteration of the myelin structure 20. AhR deficiency also provokes demyelinating disease and inflammation 21. Several non-toxic AhR ligands has been reported to have neuroprotective effects. Indole derivatives such as indole 3-carbinol (I3C) effectively inhibit amyloid fibrillation 22. The natural flavonoid substance diosmin could rescue scopolamine-induced synaptic plasticity and cognitive impairment 23. Another study showed that oral treatment with diosmin reduced cerebral Aβ levels, tau hyperphosphorylation, neuroinflammation and cognitive impairment in the 3×Tg-AD mice 24. Interestingly, kynurenic acid induced the expression and activity of NEP in human neuroblastoma SHSY5Y cells and in mouse cortical neuron cultures 25. Intraperitoneal injection of the AhR agonist β-naphthoflavone has been reported to upregulate AhR target gene expression in the brain, including cytochrome oxidase and various antioxidant enzymes 26. Therefore, we explored whether AhR directly regulates the expression of ADEs in the brain and whether it plays a role in Aβ metabolism. Furthermore, we examined the relationship between AhR and NEP. We found that AhR functions as a transcription factor of NEP and demonstrated that both endogenous and exogenous ligands effectively increased the expression and activity of NEP *in vitro* and *in vivo*. The effect of AhR ligands on ameliorating learning and memory deficiency in the APP/PS1 mouse model was also confirmed.

## Material and Methods

### Materials

Anti-AhR antibody (Cat No.: 17840-1-AP) was purchased from Proteintech (Wuhan, China). Anti-NEP antibody (Cat No.: sc-46656) was purchased from Santa Cruz (Dallas, TX, USA). Anti-β-actin antibody (Cat No.: AF5001), HRP-labeled donkey anti-rabbit IgG (Cat No.: A0208) and HRP-labeled donkey anti-mouse IgG (Cat No.: A0216) were purchased from Beyotime Biotechnology (Shanghai, China). Anti-NeuN (Cat No.: ab177487), anti-GFAP (Cat No.: ab7260) and anti-IBA1 (Cat No.: ab178847) antibodies were purchased from Abcam (Cambridge, MA, UK). Alexa Fluor® 488 AffiniPure donkey anti-mouse IgG (H+L) (Code No.: 715-545-150) and Cy™3 AffiniPure donkey anti-rabbit IgG (H+L) (Code No.: 711-165-152) were purchased from Jackson ImmunoResearch (West Grove, PA, USA). The mouse NEP enzyme linked immunosorbent assay (ELISA) kit (Cat No.: CSB-EL014653MO), human Aβ42 ELISA kit (Cat No.: CSB-E10684h) and human Aβ40 ELISA kit (Cat No.: CSB-E08299h) were purchased from Cusabio (Wuhan, China). Diosmin (CAS No.: 520-27-4, Cat No.: HY-N0178), FICZ (CAS No.: 172922-91-7, Cat No.: HY-12451), Indole-3-acetamide (I3C) (CAS No.: 879-37-8, Cat No.: HY-W016784) and Sacubitril (Sac) (CAS No.: 149709-62-6, Cat No.: AHU-377) were purchased from MedChemExpress (Monmouth Junction, NJ, USA). StemRegenin 1 (SR1) (CAS No.: 1227633-49-9, Cat No.: S2858) was purchased from Selleckchem (Shanghai, China).

### Cell culture

Neuro-2a (N2a) (Cat No.: ZQ0207) and HEK293T (Cat No.: ZQ0033) cells were obtained from Zhong Qiao Xin Zhou Biotechnology (Shanghai, China). The cells were authenticated using the short tandem repeat (STR) method. N2a cells were cultured in Eagle's Minimum Essential Medium (EMEM) (ATCC, Manassas, VA, USA) supplemented with 10% (v/v) fetal bovine serum (FBS) (Gibco, Grand Island, NY, USA), 100 μg/mL streptomycin and 100 units/mL penicillin (GE Healthcare Hyclone, Chicago, IL, USA). HEK293T cells were cultured in Dulbecco's Modified Eagle Medium (DMEM) (Keygen, Nanjing, China) supplemented with 10% (v/v) fetal bovine serum (FBS) (Gibco), 100 μg/mL streptomycin and 100 units/mL penicillin (Hyclone). Cells were cultured at 37 ºC with 5% (v/v) CO_2_.

### Small interfering RNA knockdown and plasmid transfection

To knockdown of AhR, N2a cells were transfected with siRNA or scrambled siRNA (Santa Cruz, sc-29655) using Lipofectamine 3000 (Invitrogen, Carlsbad, CA, USA) according to the manufacturer's protocol. The cells were used in subsequent experiments 48 h after transfection.

To generate an Aβ42 overexpression cell line, we transfected N2a cells with pcDNA FRT TO-APPSwed/Ind (Addgene plasmid, Cat No. #114194). After 24 h, the culture medium was switched to a complete growth medium with 200 μg/mL hygromycin B (Roche, Basel, Switzerland). Individual colonies were isolated, and the soluble Aβ42 levels in the culture medium were determined by ELISA (Cusabio).

### Animal study

All animal experiments were approved by the intramural Ethics Committee on Animal Studies and performed in accordance with the National Institutes of Health Guidelines for the Care and Use of Laboratory Animals (SYXK(SU) 2016-0011). Eight-month-old APPswe/PS1Δ9 (APP/PS1) transgenic mice [B6C3-Tg (APPswe, PSEN1dE9)85Bdo/J], weighting 35-40 g, and their wild type (WT) littermates were obtained from Hangzhou Ziyuan Laboratory Animal Technology Co., Ltd (Hangzhou, Zhejiang, China). Animals were kept under a consistent temperature (24 ºC) with a 12-h light/dark cycle and fed standard food pellets with access to sterile water *ad libitum*. APP/PS1 mice were randomly divided into four groups (n = 6-10 per group) and received daily oral administration with vehicle or diosmin at the indicated dose for 4 weeks.

### Adeno-associated virus (AAV)-mediated RNAi in the mouse brain

Mice were anaesthetized by intraperitoneal injection of pentobarbital sodium (50 mg/kg), and then placed on a stereotaxic apparatus. AAV9 vectors carrying shGFP or shAhR (8 ×10^10^ vector genomes, GenePharma, Shanghai, China) were bilaterally injected into the lateral cerebral ventricle (anteroposterior to the bregma, -0.6 mm; lateral, 1.2 mm; and ventral, 2.2 mm). The shAhR sequence: 5′-CCGGCATCGACATAACGGACGAAATCTCGAGATTTCGTCCGTTATGTCGATGTTTTTG-3′.

### Western blot assay

After the object recognition test, animals were sacrificed and perfused with cold PBS. The brains were immediately removed from the skull and the cortex and hippocampus were separated by microdissection. Tissue samples were snap-frozen in liquid nitrogen and stored at -80 °C. For the western blot assay, cells or brain tissues were washed twice with ice-cold phosphate-buffered saline (PBS) and lysed in RIPA buffer with freshly added PMSF (Beyotime). The protein concentration was measured by the BCA method using the Pierce™ BCA Protein Assay Kit (Thermo Fisher Scientific, Waltham, MA, USA). Each protein sample containing 20 μg was heated in 5×SDS loading buffer (Beyotime) at 98 ºC for 10 min. An equal amount of protein samples were run on 10 % SDS-PAGE gels and transferred to PVDF membranes (Millipore, Billerica, MA, USA). The membranes were then blocked with 5% non-fat milk (BD Difco, Detroit, MI, USA) and immunoblotted with anti-AhR (1:1000), anti-NEP (1:1000) or anti-β-actin (1:5000) primary antibodies followed by HRP-labeled goat anti-rabbit or anti-mouse secondary antibodies (1:5000). The protein bands were detected by Chemistar™ High-sig ECL Western Blotting Substrate (Tanon, Shanghai, China) and imaged with the ChemiDoc XRS+ system (Biorad, Hercules, CA, USA). Images were quantified using the NIH ImageJ v1.53c software (Bethesda, MD, USA). After opening the images in ImageJ, certain bands were circled and the intensities were measured. The protein expression levels were adjusted to β-actin, which was used as the loading control.

### ELISA

Brain NEP and detergent-soluble Aβ42 and Aβ40 was detected by ELISA kits. To determine the NEP level, about 20 mg of brain tissue was homogenized in 1 mL of PBS. The homogenates were centrifuged for 5 min at 5000 ×g. The supernatant was collected and immediately assayed following the manufacturer's instructions or stored at -80 ºC. The NEP levels were normalized by the total protein concentration.

Soluble Aβ42 and Aβ40 was extracted as described previously 27; frozen tissues were homogenized in cold lysis buffer (0.2 % diethylamine and 50 mM NaCl) and centrifuged at 100,000 × g for 1 h at 4 ºC. The supernatant was collected and neutralized by adding 0.1 × volume of 0.5 M Tris HCl (pH 6.8). The neutralized samples containing soluble Aβ42 and Aβ40 were analyzed by ELISA or flash-frozen on dry ice and stored at -80 ºC.

### Quantitative RT-PCR

Total RNA was extracted from cells or brain tissues using RNA-easy Isolation Reagent (Vazyme, Nanjing, Jiangsu, China) and reverse transcribed into cDNA using a HiScript Reverse Transcriptase kit (Vazyme). Gene expression was determined by the QuantStudio Q5 System (Applied Biosystems, Waltham, MA, USA) using Hieff® qPCR SYBR Green Master Mix (Yeasen, Shanghai, China). Gene expression was normalized to *Gapdh* expression. The sequences of primers used in the experiments are listed in Table 1.

### Immunofluorescence

Formaldehyde-fixed frozen brain sections were blocked with blocking buffer (PBS, 0.3 % Triton X-100, 5% normal donkey serum) and then incubated with the indicated primary antibodies at 4 ºC overnight. Tissue slides were then incubated with Alexa Fluor 488® or Cy™3 AffiniPure secondary antibodies for 1 h at room temperature. All primary and secondary antibodies were diluted in antibody dilution buffer (PBS, 0.3% Triton X-100, 1% BSA). The primary antibodies were used at 1:500 dilution, except for the anti-NEP antibody, which was used at 1:50 dilution. The secondary antibodies were used at 1:500 dilution. Tissue slides were subsequently incubated with DAPI (Thermo Fisher Scientific) for 15 min and washed with PBS before being mounted with a fluorescence mounting medium (KeyGen, Nanjing, China). Immunofluorescence was visualized and captured by a confocal microscope (LSM 700, Zeiss, Göttingen, Germany). The NEP intensities were quantified using the NIH ImageJ v1.53c software (Bethesda) as mentioned before 28. After opening the images in ImageJ, the specific stained area was selected by setting the threshold. The background signal was excluded through this procedure. The signal intensity of the selected area was then measured. The colocalization of NEP and NeuN, Iba-1, or GFAP was analyzed using Pearson's correlation coefficient through ImageJ as described before 29, 30.

### Morris water maze test

The Morris water maze (MWM) test was performed as previously described 31, 32. In brief, a circular tank (1.2 m in diameter) was divided into four quadrants, and a transparent cylinder platform (8 cm in diameter) was fixed in the center of the quadrant IV, 0.5 cm under the water. Quadrant IV was considered the target quadrant. Animals were individually released into the water randomly in each quadrant at the water level, facing the tank wall, and allowed to freely swim for 90 s to find the hidden platform. If the mice failed to find the platform within the allotted time, they were guided and placed on the platform for 15 s. The spatial acquisition trial was conducted over 5 days, with four trials per day. The interval between trails was 30 min. A spatial probe trial was performed 24 h after the last acquisition day. In this trail, the platform was removed and the animals were individually released into the pool from quadrant II. Each mouse was allowed to swim for 60 s to search the platform. The escape latency, swimming speed, time spent in the target quadrant and the number of platform crossings were recorded and analyzed by an analysis management system (Viewer 2 Tracking Software, Ji Liang Instruments, China).

### Object recognition test

The object recognition test was performed as previously described 33, 34. Consider the gender differences in the behavioral response to spatial and object novelty in mice, only male mice were subjected to this test 35. The experimental design is shown in Figure 6H. Briefly, the test was performed in a square (50-cm side length) open field apparatus. Animals were first individually placed in the empty open field for 5 min to habituate them to the environment. The familiarization trail was performed 24 h after the habituation session. Two identical white cubes (8-cm side length) were placed in the open field, 5 cm away from the wall. Animals were individually placed in the apparatus and allowed to freely explore for 10 min. Twenty-four hours later, the test session was performed. The two familiar objects were replaced, one with a third cube copy and one with a blue cylinder with a diameter and height of 10 cm. The cylinder acted as the new object in the present experiment. Then, animals were individually allowed to explore for 2 min. The exploration time of the familiar object and the novel object was recorded for analysis (Viewer 2 Tracking Software). The discrimination index was calculated as follows: Discrimination index = (% time with novel object - % time with familiar object)/(% time with novel object + % time with familiar object).

### NEP enzymatic activity assay

NEP enzymatic activity assay was performed with the Neprilysin Activity Assay Kit (Fluorometric) (BioVision, Milpitas, CA, USA). Cells or brain tissues were lysed in ice-cold NEP Assay Buffer containing 1 mM PMSF (Beyotime) and 10 μg/mL Aprotinin (Sigma, A1153). Protein concentrations were measured by using the BCA Protein Assay Kit (Beyotime, China). Then, 10 μg of total protein was placed in the wells of a 96-well white opaque plate before adding the substrate working solution. Reagents were mixed by shaking the plate gently for 30 s, and the fluorescence signal was immediately measured at Ex/Em = 330/340 nm on the kinetic mode at 37 ºC. The data were recorded every 5 min for 1 h. The NEP-specific activity is expressed as U/mg of protein.

### Luciferase reporter assay

The *NEP* promoter-luciferase plasmid was constructed by cloning a fragment of the human *NEP* promoter region into the pGL3-basic luciferase vector using the KpnI and NcoI restriction sites. The WT AhR expression plasmid was obtained by cloning WT human *AHR* cDNA into a pBind-ΔDBD plasmid. HEK293T cells were grown to 80% confluency in 24-well plates. Then, the *NEP* promoter-luciferase plasmid (250 ng) and AhR expression plasmid (250 ng) were co-transfected into cells using Lipofectamine 3000 according to the manufacturer's protocol. Twenty-four hours after transfection, cells were treated with the AhR agonists, diosmin (80 μM), FICZ (250 nM), I3C (10 μM) and L-KN (3 μM) with or without SR1 (2 μM) (MedChemExpress) for 18 h. The luciferase activity was measured by the Dual-Lumi II Luciferase Gene Reporter Gene Assay Kit (Beyotime).

### Chromatin immunoprecipitation (ChIP)

The ChIP assay was performed with the SimpleChIP Plus Enzymatic Chromatin IP Kit (Cell Signaling Technology, Danvers, MA, USA) following the manufacturer's recommendations. Precipitated genomic DNA was amplified by real-time PCR with the following primers: site1: forward: 5′-GGCGCATTACGGCTGATTTC-3′; reverse: 5′-AACTGAAACTCTCCGCTCCC-3′; site2: forward: 5′- TATAGTCTCCGGTCCACAGGG-3′; reverse: 5′- TTGCTTAGCTTCGGGGCTTT-3′.

### Statistical analyses

All data are expressed as mean ± SD. Comparisons between two groups were performed using Student's *t*-test, and comparisons between three or more sets of data were performed using one-way ANOVA followed by Dunnett's *pos hoc* test. Differences with *p* < 0.05 were considered statistically significant.

## Results

### Activated AhR increases NEP expression in neurons

**Figure 1:**
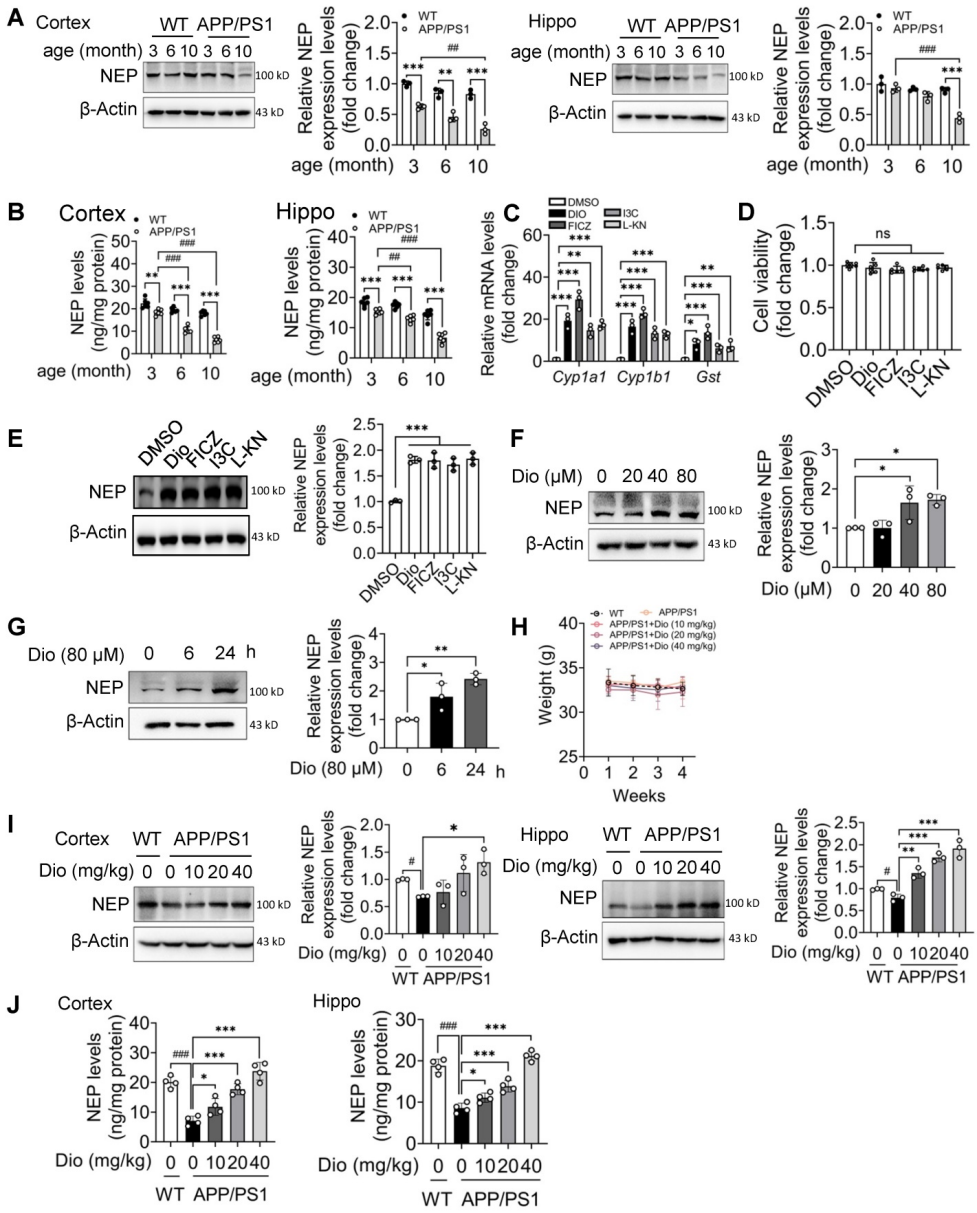
** Activated AhR increases NEP expression in N2a cells and in the brain of APP/PS1 mice. (A and B)** The cortex and hippocampus tissues from WT mice and APP/PS1 mice at the indicated age were collected, and homogenized, and the NEP levels were determined by western blot analysis (n = 3) (A) and ELISA (n = 6) (B). NEP expression was adjusted to β-actin, which was used as the loading control in western blot analysis (A) or normalized to the total protein in ELISA (B). **(C)** N2a cells were treated with DMSO or Dio (40 µM) for 6 h, the total mRNA was extracted and the mRNA levels of *Cyp1a1*, *Cyp1b1* and *Gst* were determined by qRT-PCR, n = 3. **(D)** N2a cells were treated with DMSO, Dio (80 μM), FICZ (250 nM), I3C (10 μM) or L-KN (3 μM) for 24 h and the cell viability was measured by the CCK8 assay. n = 6. **(E)** N2a cells were treated with DMSO, Dio (40 μM), FICZ (250 nM), I3C (10 μM) or L-KN (3 μM) for 12 h. Whole cell extracts were examined by western blotting with the indicated antibodies. NEP expression was adjusted to that of β-actin, which was used as the loading control. n = 3. (F and G) N2a cells were treated with Dio at the indicated concentrations for 12 h (n = 3) **(F)** or with 80 μM Dio for the indicated time (n = 3) **(G).** The NEP protein levels were determined by western blotting. Eight-month-old APP/PS1 mice were treated with vehicle or Dio at the indicated concentration for 4 weeks. **(H)** The bodyweight was monitored during this period. At the end of the treatment, the mice were sacrificed and the cortex and hippocampus tissues were collected. The NEP protein levels were determined by western blot analysis (n = 3) **(I)** or ELISA (n = 4) **(J).** Data were expressed as mean ± SD. # *p* < 0.05; ## *p* < 0.01, ### *p* < 0.001 vs. indicated group, * *p* < 0.05, ** *p* < 0.01, *** *p* < 0.001 vs. indicated group. Dio: diosmin; Hippo: hippocampus; I3C: indole-3-carbinol; L-KN: L-Kynurenine;NEP: neprilysin;WT: wild type.

Compared with WT mice at the same age, the NEP protein levels in the cortex and hippocampus of the APP/PS1 mice were lower and further decreased with age (Figure 1A, B). Because in the brain, the NEP is mainly expressed in neurons (Figure S1), we chose the mouse neuron cell line N2a cells for further study. To determine the effects of AhR agonists on NEP expression, we first treated N2a cells with the exogenous AhR agonist diosmin or I3C, or with the endogenous AhR agonist FICZ or L-KN. AhR activation after treatment with these compounds at the indicated concentrations was confirmed by checked the mRNA levels of AhR target genes in N2a cells, including *Cyp1a1, Cyp1b1,* and *Gst* 26 (Figure 1C). All the test compounds showed negligible cytotoxicity in N2a cells (Figure 1D). As shown in Figure 1E, the AhR agonists significantly induced NEP expression in N2a cells. Furthermore diosmin, a marketed medicine, promoted NEP expression in a dose (Figure 1F) and time-dependent (Figure 1G) manner.

**Figure 2:**
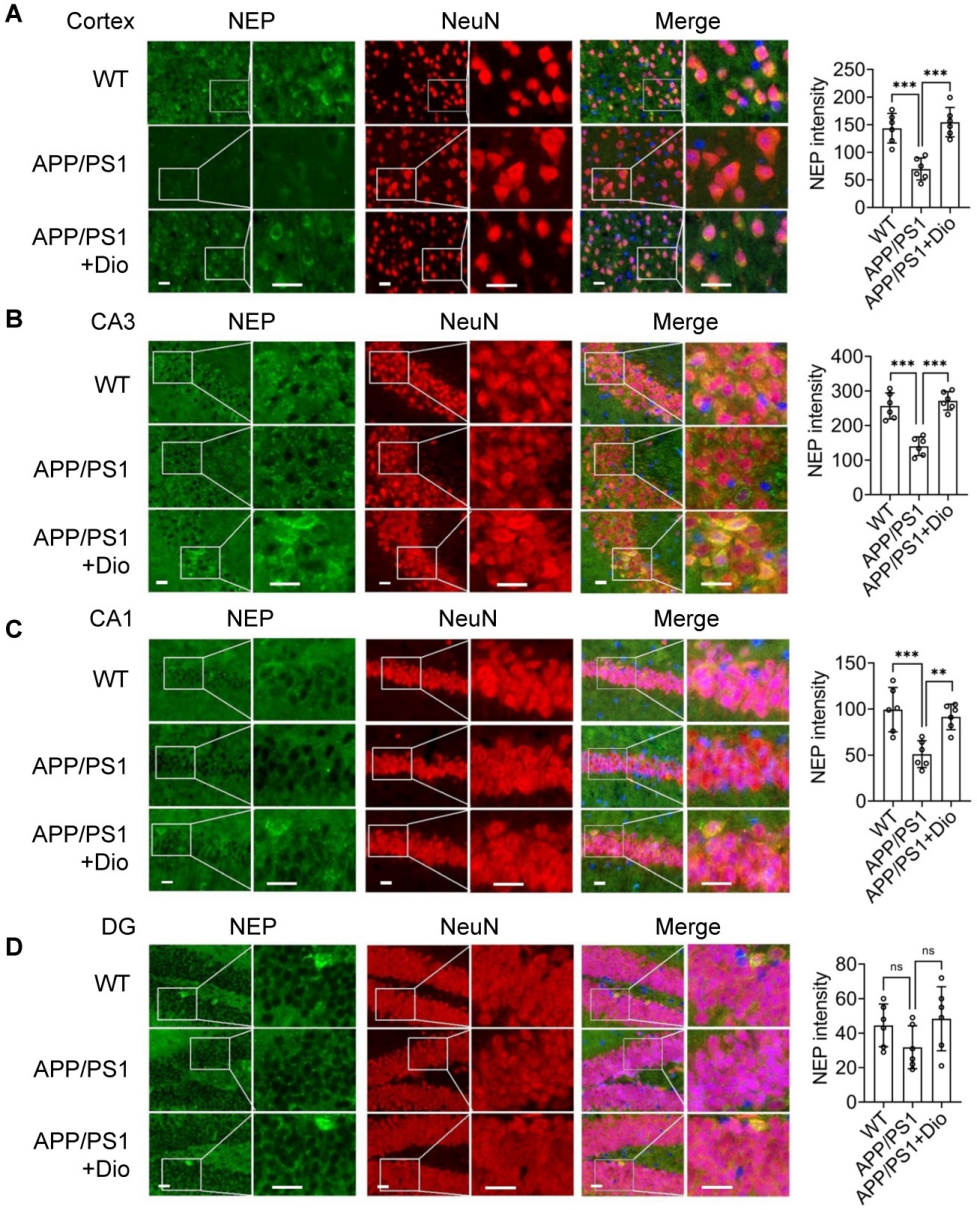
** Activated AhR increases NEP expression in the brain of APP/PS1 mice.** Eight-month-old APP/PS1 mice were treated with vehicle or Dio at the indicated concentration for 4 weeks. At the end of the treatment, the mice were sacrificed and the brain tissues were fixed and stained for NEP (green), NeuN (red) and DAPI (blue). NEP staining and quantification in cortex **(A)**, and CA3 **(B)**, CA1 **(C)** and DG **(D)** of the hippocampal regions. Scale bar: 20 μm. n = 6. Each point represents the mean intensity of NEP for 3-5 sections per mouse. Data were expressed as mean ± SD. ** p < 0.01, *** *p* < 0.001 vs. APP/PS1 group. CA: Cornus Ammonis; DG: dentate gyrus; Dio: diosmin; NEP: neprilysin;WT: wild type.

We then investigated the *in vivo* effect of diosmin on APP/PS1 transgenic mice. Eight-month-old male (Figure 1H-J) and female (Figure S2) APP/PS1 mice were treated with diosmin or vehicle for 4 weeks. The body weight of the mice in different groups was not significantly altered during treatment (Figure 1H, Figure S2A). However, the NEP protein levels in the cortex and hippocampus (Figure 1I-J, Figure S2B) of the APP/PS1 mice were increased by the diosmin treatment compared with the vehicle control. We further performed immunofluorescence staining analysis. NEP immunoreactivity in the diosmin treated group was significantly elevated in the neuron cell bodies in the cortex (Figure 2A), cornus ammonis 3 (CA3) (Figure 2B), CA1 (Figure 2C), and dentate gyrus (DG) (Figure 2D) of the hippocampal regions compared with the APP/PS1 group.

**Figure 3:**
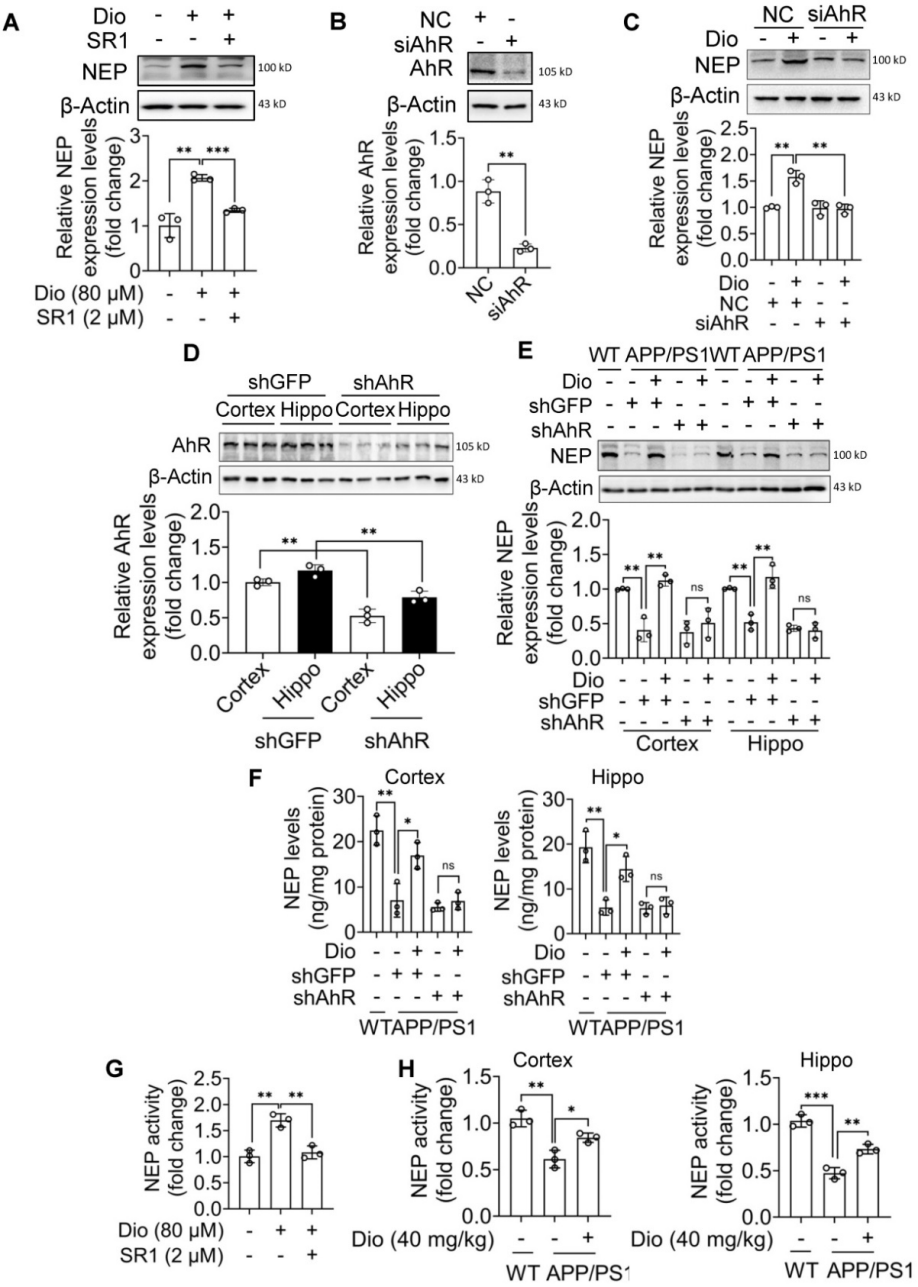
** Diosmin-induced NEP expression depends on activate AhR. (A)** N2a cells were treated with Dio (80 μM) with or without SR1 (2 μM) for 12 h. Whole cell lysates were collected and the NEP levels were determined. n = 3. **(B)** N2a cells were transfected with non-target control siRNA (NC) or siRNA targeting AhR (siAhR) for 48 h. The AhR levels were determined by western blotting. n = 3. **(C)** N2a cells were transfected with NC or siAhR for 48 h, followed by administration of vehicle or Dio (80 μM) for 12 h. NEP levels were then determined by western blotting. n = 3. APP/PS1 mice (8 months of age) received an intracerebroventricular injection with AAV9 carrying the shRNA targeting AhR (shAhR) or the shRNA targeting GFP (shGFP). Five days after injection, the mice were administrated with vehicle or Dio (40 mg/kg) for another 4 weeks. Total protein from the cortex and hippocampus tissues was prepared to determine the AhR levels by western blotting **(D)** and the NEP levels by western blotting **(E)** and ELISA **(F)**, n = 3. **(G)** N2a cells were treated with Dio (80 μM) with or without SR1 (2 μM) for 12 h. n = 3.** (H)** APP/PS1 mice (8 months of age) were administrated with vehicle or Dio (40 mg/kg) for 4 weeks. Whole cell lysates of cells and animal tissues were collected. n = 3. The NEP enzyme activity in the samples was detected by a NEP Activity Assay Kit. Data were expressed as mean ± SD. * *p* < 0.05, ** *p* < 0.01 and *** *p* < 0.001 vs. indicated group. Dio: diosmin; NC: nontarget control; NEP: Neprilysin; SR1: StemRegenin 1.

SR1 is a small molecular compound that can directly bind to AhR to inhibit its activity 36. As shown in Figure 3A, SR1 significantly blocked the diosmin effect in N2a cells. Similarly, after AhR knockdown by siRNA, diosmin treatment did not increase the NEP protein levels in N2a cells (Figure 3B, C). We then investigated the *in vivo* effect of diosmin on NEP expression after knocking down AhR in the brain. The AAV delivery into the CSF through ICV injection has been shown to achieve efficient gene transfer to a broad area of the brain in adult mice 37, 38. Furthermore, AAV9 has been reported to effectively targets neurons in adult mice through single stereotaxic or intracardiac injection 39, 40. In the present study, AAV9 carrying shRNA targeting *AhR* (shAhR) or *GFP* (shGFP) was injected into at 8-month-old APP/PS1 mice *via* lateral ventricles, leading to a significant decrease in the cortex and hippocampus AhR expression (Figure 3D). To rule out the possible adverse effects of AhR knockdown, we treated WT mice (8 months of age) with shGFP or shAhR in the same method and then subjected them to MWM and object recognition test. Results showed that AhR knockdown through AAV9 injection did not significantly alter the cognition of mice (Figure S3A-F). The *in vivo* effect of diosmin on NEP expression was also abolished by AhR knockdown (Figure 3E-F, Figure S3G-H).

To confirm that diosmin activation of AhR induces functional NEP expression, we examined the NEP enzyme activity in N2a cells and brain tissue. As shown in Figure 3G, diosmin and the other AhR agonists (Figure S4A-C) treatment effectively increased the NEP enzyme activity *in vitro*. Oral administration of diosmin also increased NEP activity in the brain (Figure 3H, Figure S4D-F). Moreover, diosmin failed to upregulate the NEP enzyme activity in the presence of SR1 in N2a cells (Figure 3G).

### AhR is the transcription factor of NEP

**Figure 4:**
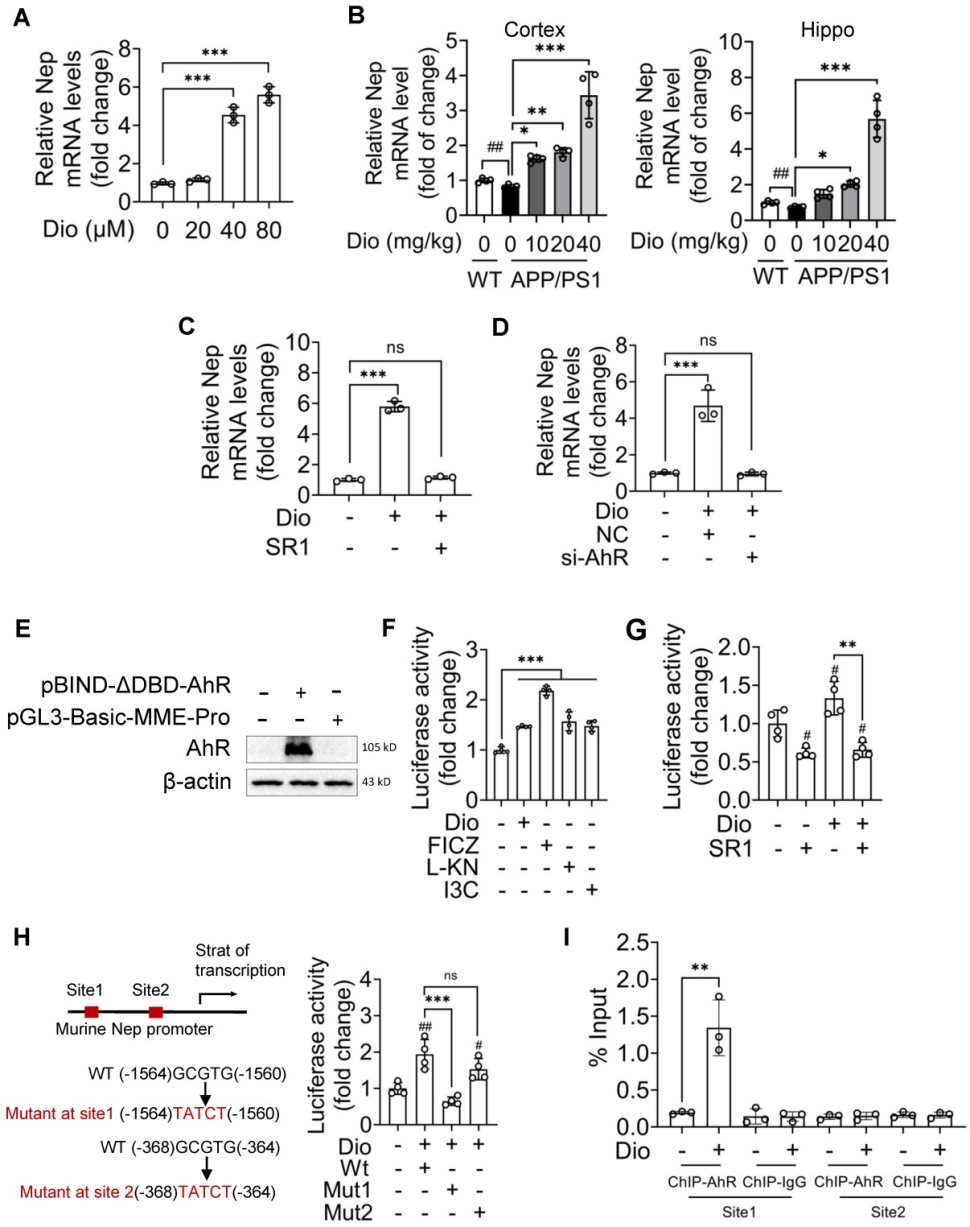
** AhR transcriptionally upregulates NEP expression. (A-D)** N2a cells were treated with Dio at the indicated concentrations for 6 h, and then the total mRNA was extracted (n = 3) (A); APP/PS1 mice (8 months of age) were administrated with vehicle or Dio (40 mg/kg) for 4 weeks. The WT littermates were used as control (B); N2a cells were treated with Dio (80 μM) with or without SR1 (2 μM) for 6 h (n = 3) (C); N2a cells were transfected with non-targeting siRNA (NC) or siRNA targeting AhR (siAhR) for 48 h, and then treated with Dio (80 μM) for another 6 h (n = 3) (D). The total RNA samples were extracted from the cell and tissue samples. The *Nep* mRNA levels were determined by qRT-PCR. n = 3. **(E-G)** HEK293T cells were co-transfected with *NEP* promoter-luciferase plasmid and WT AhR expression plasmid for 48 h. Whole cell lysates were collected and the AhR expression levels were determined by western blotting (E). After transfection, HEK293T cells were treated with Dio (80 μM), FICZ (250 nM), L-KN (3 μM) or I3C (10 μM) for another 18 h (n = 4) (F), or with Dio (80 μM) with or without SR1 (2 μM) for 18 h (n = 4) (G). Whole cell lysates were collected and the luciferase activity was determined. **(H)** HEK293T cells were co-transfected with WT AhR expression plasmid and WT or mutant (site1 or site2) *NEP* promoter-luciferase plasmid for 48 h. The cells were then treated with Dio (80 μM) for an additional 18 h, and then the luciferase activity was determined. n = 4. **(I)** ChIP-qPCR analysis of AhR binding to the *NEP* promoter in N2a cells. n = 3. Data were expressed as mean ± SD. ## *p* < 0.01 vs. WT treated with vehicle or N2a cell treated with DMSO, * *p* < 0.05, ** *p* < 0.01 and *** *p* < 0.001 vs. indicated group. Dio: diosmin; I3C: indole-3-carbinol; L-KN: L-Kynurenine; Mut: mutant; SR1: StemRegenin 1; WT: wild type.

To explore the role of AhR in NEP induction, we investigated the effect of AhR agonists on the transcription levels of NEP. In N2a cells, diosmin (Figure 4A), as well as the other tested AhR agonists (Figure S4D-F), induced *Nep* gene expression. Similarly, diosmin also upregulated *Nep* mRNA levels in the cortex and hippocampus of APP/PS1 mice (Figure 4B). In contrast, AhR inhibitor SR1 (Figure 4C) or siRNA against AhR (Figure 4D) diminished the diosmin-induced *Nep* mRNA expression in N2a cells, suggesting that diosmin increases NEP expression through AhR-mediated transcriptional regulation. Effects of diosmin on the transcription levels of insulin-degrading enzyme (IDE), endothelin-converting enzymes (ECE), angiotensin-converting enzyme (ACE), plasminogen were also determined (Figure S4G).

Next, we co-transfected HEK293T cells with the *NEP* promoter-luciferase plasmid and the WT AhR expression plasmid (Figure 4E). The luciferase reporter assays showed that diosmin and the other tested AhR agonists effectively increased the luciferase reporter activity of NEP (Figure 4F), while SR1 blocked it (Figure 4G). On the basis of the canonical AhR recognition motif (GCGTG) 41, we identified two potential AhR-binding sites in the *NEP* promoter region (Figure 4H). We mutated these sites separately (termed site1 and site2), and when we co-transfected the cells with AhR expression plasmid and the *NEP* promoter-luciferase plasmid mutated at site1 or site2, we found that diosmin failed to increase the luciferase activity when site1 was mutated but not when site2 was mutant. Consistently, the ChIP assay showed that diosmin induced AhR recruitment to the *NEP* gene promoter at site 1 but not at site2 (Figure 4I). These results indicated that AhR plays a critical role in *NEP* gene transcription and that it has a specific potential binding site.

### Activating AhR decreases Aβ42 levels in a NEP-dependent manner

**Figure 5:**
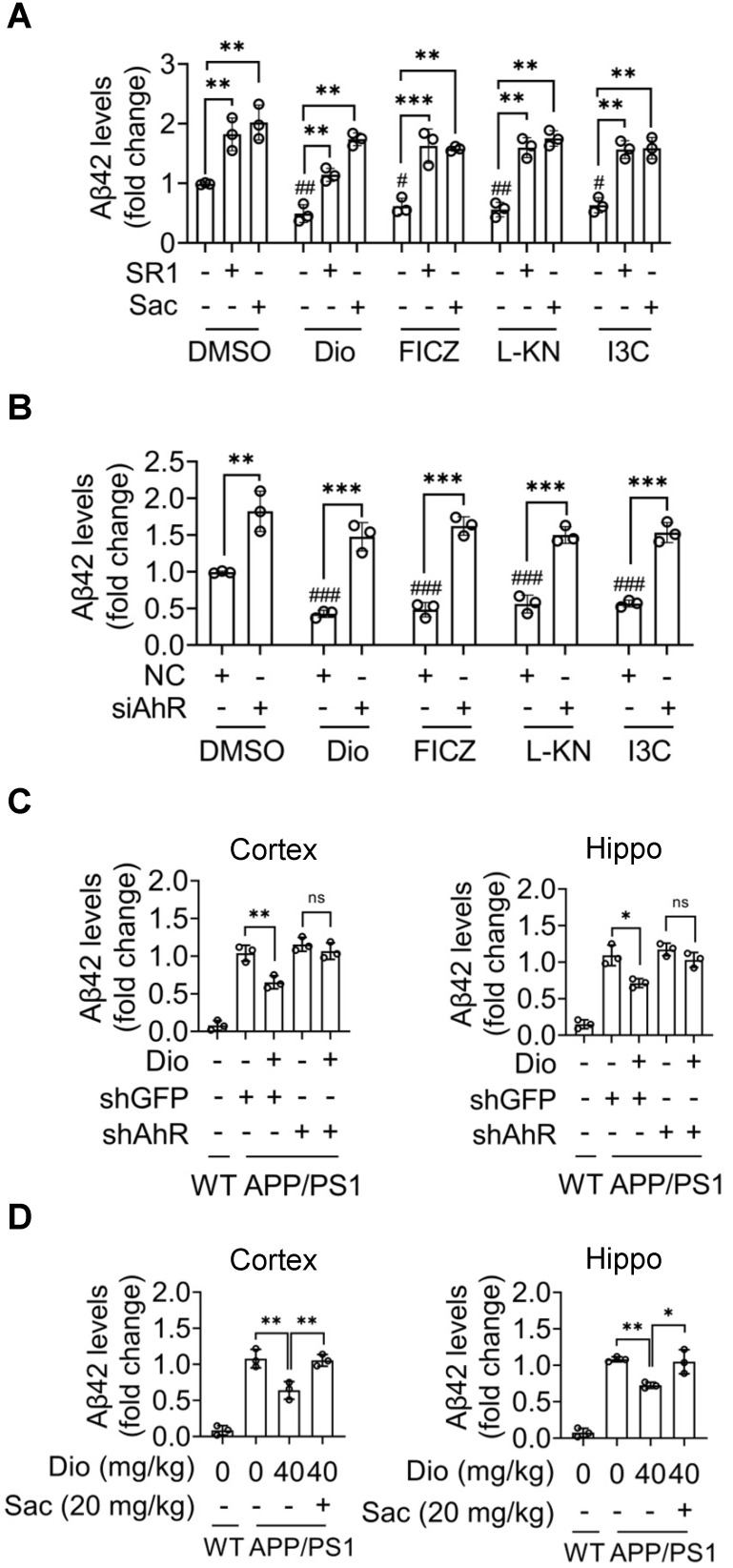
** Activated AhR decreases Aβ42 levels in a NEP-dependent manner. (A)** N2a-APP cells were treated with Dio (80 μM), FICZ (250 nM), L-KN (3 μM) or I3C (10 μM) with or without SR1 (2 μM) or Sac (1 μM) for 24 h, the supernatant was collected and the Aβ42 levels were determined by ELISA. **(B)** N2a-APP cells were transfected with non-targeting siRNA (NC) or siRNA targeting AhR (siAhR) for 48 h, and then administrated with the compounds mentioned in (A) for an additional 24 h. The soluble Aβ42 levels in the supernatant were measured by ELISA. **(C)** APP/PS1 mice (8 months of age) were subjected to ICV injection with AAV9 carrying the shRNA targeting AhR (shAhR) or the shRNA targeting GFP (shGFP). Five days after injection, animals were administrated with vehicle or Dio (40 mg/kg) for another 4 weeks. **(D)** The APP/PS1 mice (8 months of age) were administrated vehicle or with Dio (40 mg/kg) with or without Sac (20 mg/kg) for 4 weeks. At the end of treatment, the mice were sacrificed. The cortex and hippocampus tissues were collected and homogenized. The soluble Aβ42 was extracted and analyzed by ELISA. n = 3. Data were expressed as mean ± SD. # *p* < 0.05, ## *p* < 0.01, ### *p* < 0.001 vs. DMSO group; * *p* < 0.05, ** *p* < 0.01, *** *p* < 0.001 vs. the indicated group. Dio: diosmin; Hippo: hippocampus; I3C: indole-3-carbinol; L-KN: L-Kynurenine; Sac: Sacubitrilat; SR1: StemRegenin 1.

To determine the effects of AhR agonists on Aβ42 metabolism, N2a-APP cells that stably overexpression and secret Aβ42 peptide were used. The N2a-APP cells were treated with AhR agonists for the indicated durations, and the Aβ42 levels in the supernatants were determined. Administration of exogenous and endogenous AhR agonists resulted in a significant decrease in the concentration of Aβ42 in the supernatants (Figure 5A). In contrast, inhibition of AhR activity with SR1, or inhibition of NEP with Sac (Figure 5A), or AhR knockdown (Figure 5B) abolished the AhR agonists' effect. Consistently, after AhR was knocked down *in vivo*, diosmin treatment did not alter the levels of Aβ42 in the cortex and hippocampus (Figure 5C). Moreover, NEP inhibition with Sac reversed the *in vivo* effect of diosmin (Figure 5D). Consider the ability of NEP to bind and degrade Aβ40, the levels of the soluble Aβ40 in the cell culture medium and brain tissues were analyzed. As shown in Figure S5, AhR agonists also reduced the soluble Aβ40 levels *in vitro* (Figure S5A-B) and *in vivo* (Figure S5C-D). Moreover, the AhR inhibitor (Figure S5A), NEP inhibitor (Figure S5A, D), and AhR knockdown (Figure S5B-C) blocked the effect of diosmin on Aβ40.

### Activating AhR ameliorates cognitive deficiency in APP/PS1 mice

**Figure 6:**
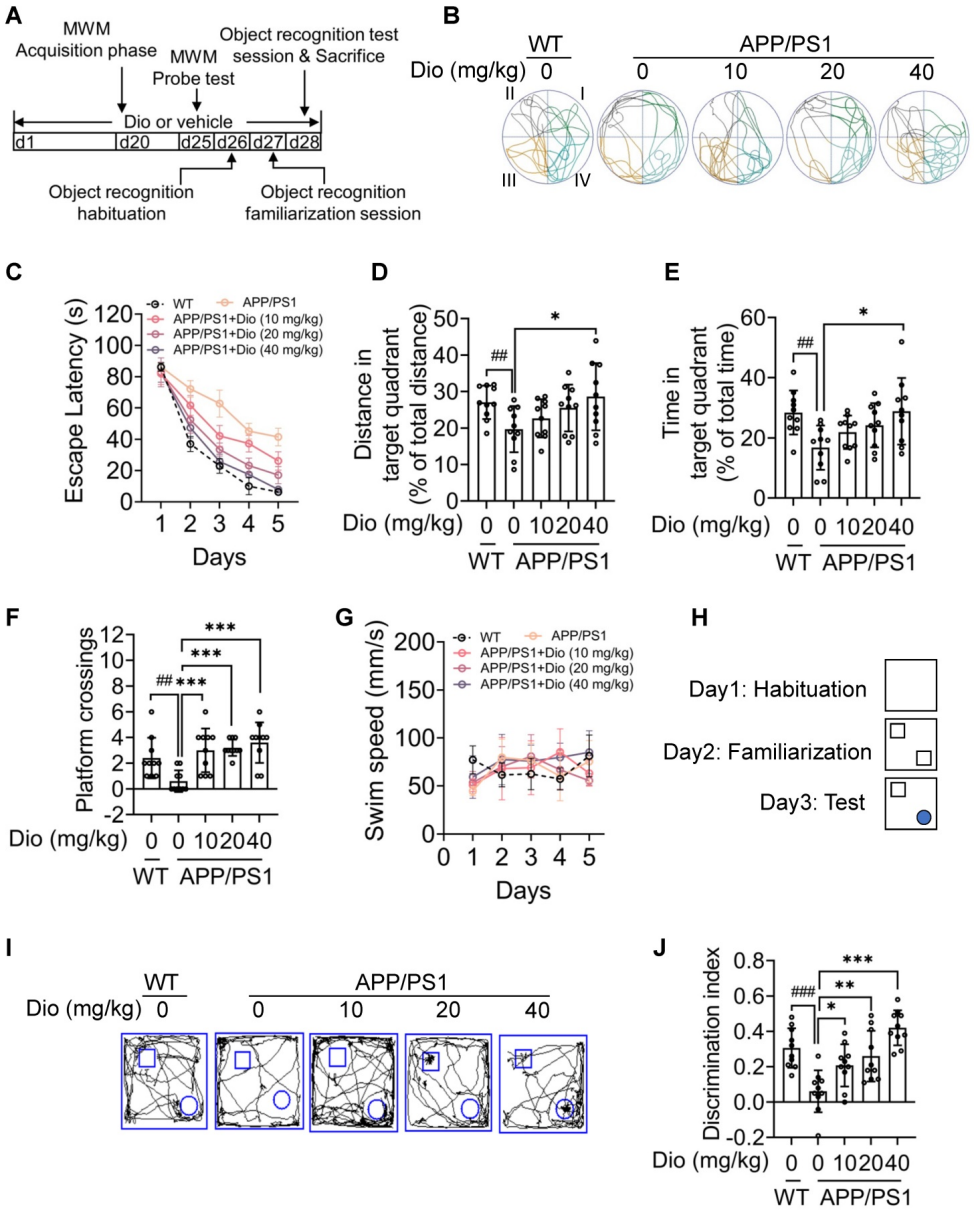
** Diosmin ameliorates cognitive deficiency in APP/PS1 mice through the AhR-NEP pathway. (A)** The experimental design of the mouse study. The APP/PS1 mice (8 months of age) were administrated vehicle or with Dio (40 mg/kg) for 4 weeks. On day 20 (d20), the mice were subjected to MWM assay. Quadrant IV was defined as the target quadrant. **(B)** Escape latency during spatial acquisition training. **(C)** Representative motion track during the spatial probe test. **(D)** Distance in the target quadrant, **(E)** time spent in the target quadrant, and **(F)** the number of platform crossings in the spatial probe test. **(G)** Swim speed during the spatial probe test. After the MWM assay was completed, the mice were subjected to the object recognition test. **(H)** The procedure for the object recognition test. The square frame represents the open field, the small square and circle represent the object. **(I)** Representative motion track and **(J)** the discrimination index of the object recognition test. n = 10. Data were expressed as mean ± SD. ## *p* < 0.01, ### *p* < 0.001 vs. WT mice treatment with vehicle, * *p* < 0.05, ** *p* < 0.01, *** *p* < 0.001 vs. APP/PS1 mice treatment with vehicle. Dio: diosmin; MWM: Morris water maze; WT: wildtype.

**Figure 7:**
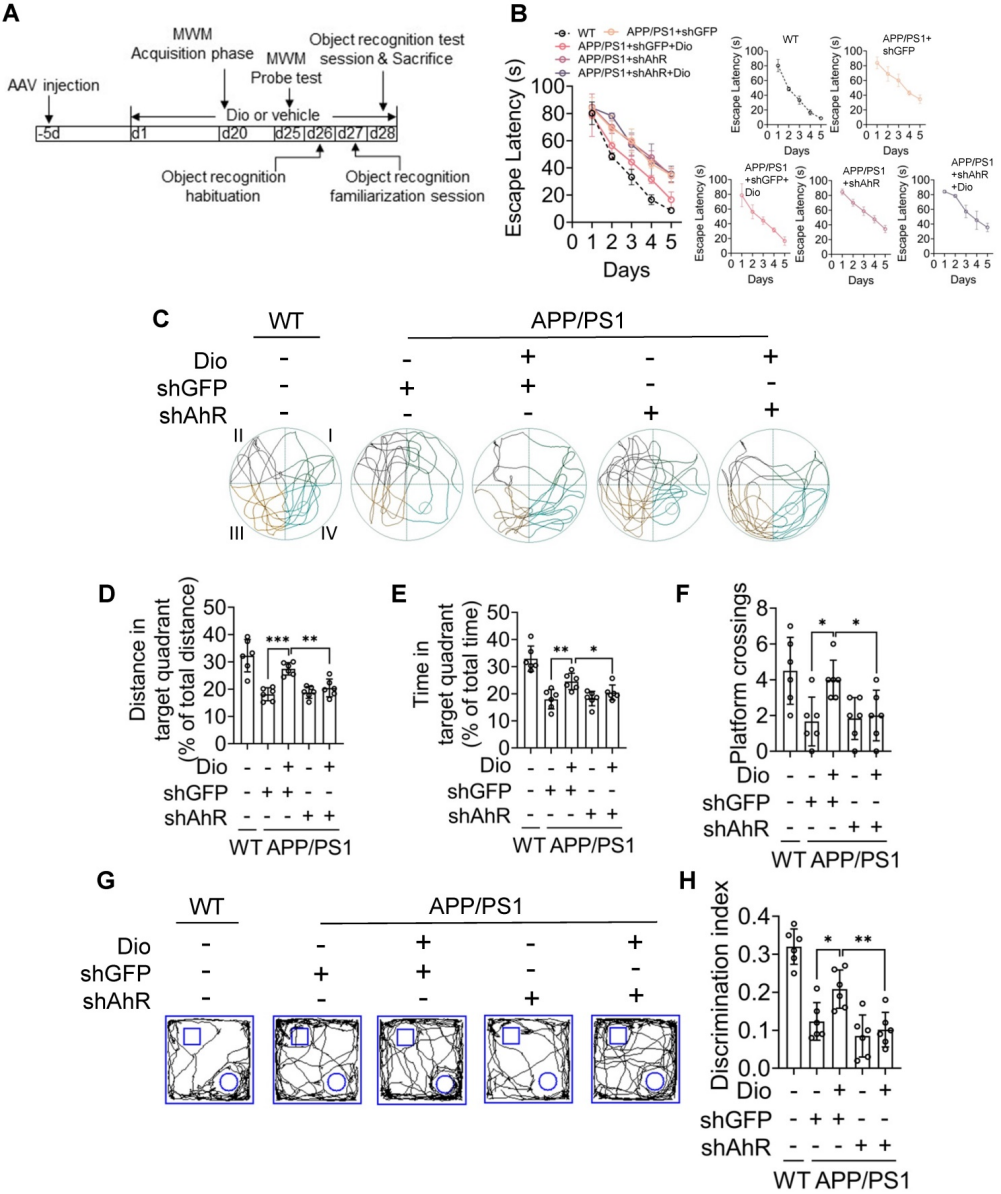
** Diosmin ameliorate cognitive deficiency in APP/PS1 mice through active AhR.** Eight-month-old WT and APP/PS1 mice were ICV injection with AAV9 carrying the shRNA targeting AhR (shAhR) or the shRNA targeting GFP (shGFP). Five days after injection, animals were administrated with vehicle or Dio (40 mg/kg) for another 4 weeks. **(A)** The experimental design of the animal study. On day 20 (d20), the mice were subjected to MWM assay. Quadrant IV was defined as the target quadrant. **(B)** Escape latency during spatial acquisition training. **(C)** Representative motion track during the spatial probe test. **(D)** Distance in the target quadrant, **(E)** time spent in the target quadrant, and **(F)** the number of platform crossing in the spatial probe test. After the MWM assay was completed, the mice were subjected to the object recognition test. The square frame represents the openfield, the small square and circle represent the object. **(G)** Representative motion track and **(H)** the discrimination index of the object recognition test. n = 6. Data were expressed as mean ± SD. * *p* < 0.05, ** *p* < 0.01, *** *p* < 0.001 vs. APP/PS1 treatment with Dio+shGFP group. AAV: adeno-associated virus; Dio: diosmin; ICV: intracerebroventricular; MWM: Morris water maze; Sac: Sacubitrilat; WT: wildtype.

**Figure 8:**
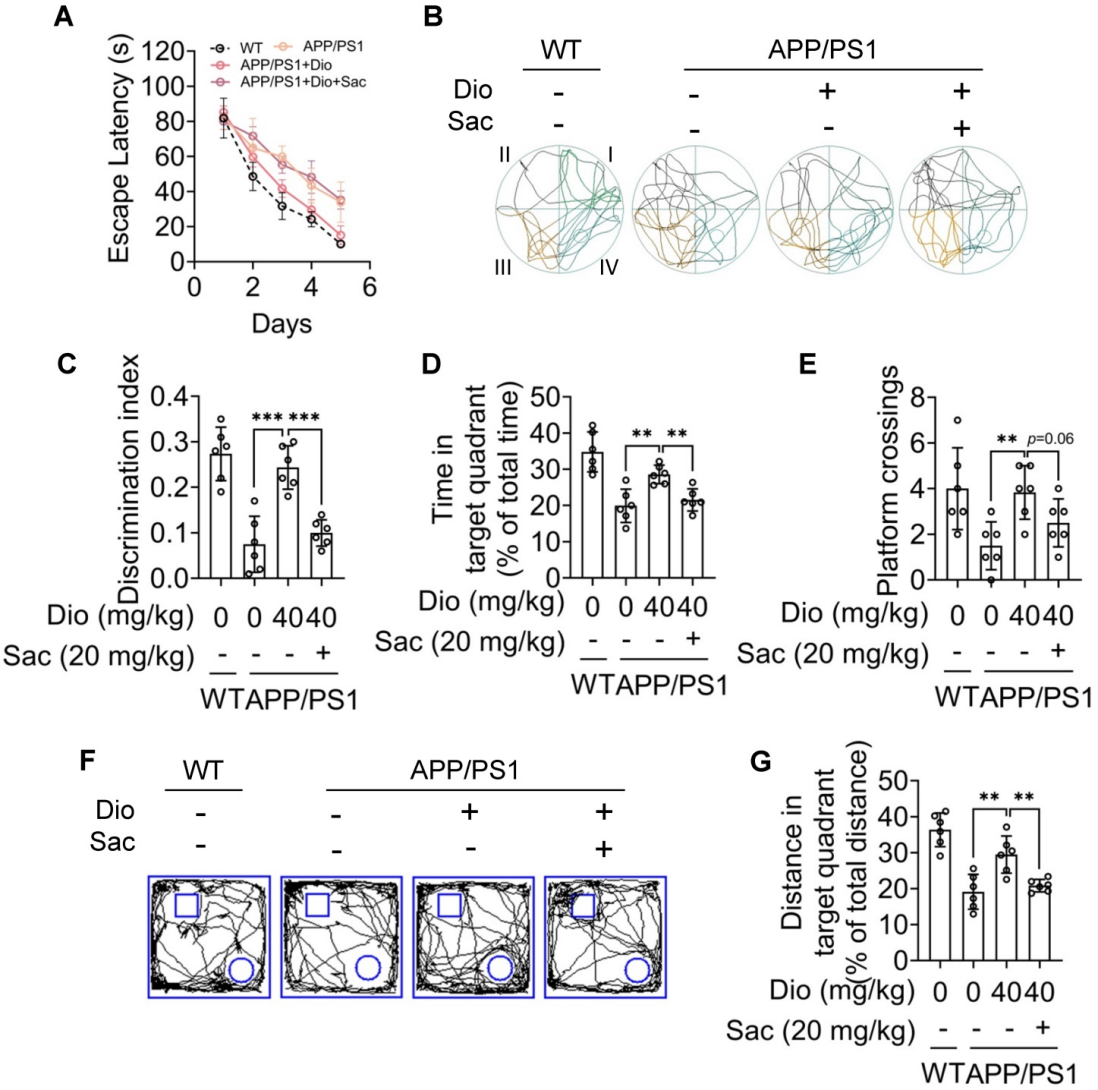
** Diosmin ameliorated cognitive deficiency in APP/PS1 mice through NEP.** Eight-month-old APP/PS1 mice were treated with vehicle, Dio (40 mg/kg) or Dio (40 mg/kg) + Sac (20 mg/kg) for 4 weeks. On day 20 (d20), the mice were subjected to MWM assay. Quadrant IV was defined as the target quadrant. **(A)** Escape latency during spatial acquisition training. **(B)** Representative motion track during the spatial probe test. **(C)** Distance in the target quadrant, **(D)** time spent in the target quadrant, and **(E)** the number of platform crossing in the spatial probe test. The square frame represents the openfield, the small square and circle represent the object. **(F)** Representative motion track and **(G)** the discrimination index of the object recognition test. n = 6. Data were expressed as mean ± SD. ** *p* < 0.01, *** *p* < 0.001 vs. APP/PS1 treatment wih Dio group. Dio: diosmin; MWM: Morris water maze; WT: wildtype.

We next evaluated whether the upregulation of NEP by AhR agonist is associated with the ameliorated cognitive deficiency of APP/PS1 mice. The MWM and object recognition test was performed to analyze the potential effects of diosmin on AD-related behavioral deficits. The scheme of the animal experiments is depicted in Figure 6A. In the MWM test, the APP/PS1 mice showed longer escape latency than the WT littermates during the spatial acquisition training, indicating a deficit in spatial learning ability (Figure 6C). While diosmin treatment improved this spatial learning deficit, the latency to reach the platform was reduced over 5 days of training (Figure 6C). In the spatial probe test, the diosmin-treated APP/PS1 mice spent more time in the target quadrant (Figure 6B, D, E) and showed a higher number of platform crossings compared with the APP/PS1 mice treated with vehicle (Figure 6F) without altered the swim speed (Figure G). However, the diosmin effect on APP/PS1 mice was blocked by AhR knockdown (Figure 7A-F) and by the NEP-specific inhibitor Sac (Figure 8A-E).

In the object recognition test, the discrimination index was used to evaluate learning and memory ability in mice, as described previously 31. As shown in Figure 6H-I, the WT mice preferred the novel objects, while the APP/PS1 mice treated with vehicle showed no significant preference. The cognitive deficiency was ameliorated by diosmin as indicated by the increased discrimination index after treatment (Figure 6J), and this effect was reversed by AhR knockdown (Figure 7G-H) or Sac treatment (Figure 8F-G). These results suggest that AhR agonist via NEP rescued the cognitive deficiency in APP/PS1 mice.

## Discussion

Aβ is usually fully catabolized immediately after secretion in young and healthy brains 5. Reduction in this catalytic process would lead to pathological accumulation of Aβ and AD development 11, 12. Several proteases have been reported to degrade the Aβ *in vitro*, while only NEP and IDE showed a significant ability to degrade extracellular and intracellular Aβ 5, 42. Manipulating these enzymes is a potential therapeutic approach to decelerate disease onset and progression 4, 15, 16, 25, 43, 44. The main toxic isoform Aβ42 can assemble into a soluble oligomer, prefibrillar, fibrillar and insoluble fibrillar plaque. Several studies have shown that the soluble Aβ42 plays a crucial role in neurotoxicity. A significant correlation was found between the levels of soluble Aβ42 and the cognitive decline in AD; therefore, targeting soluble Aβ42 may be a more effective therapeutic strategy for AD 45, 46. In the present study, we showed that diosmin treatment effectivity increased the functional NEP levels and reduced the soluble Aβ42 levels both *in vitro* and *in vivo*, suggesting this is the main mechanism of the therapeutic effect of diosmin in APP/PS1 mice. We also checked the impact of AhR agonists on other ADEs, including IDE, endothelin converting enzyme, angiotensin-converting enzyme and plasmin. As shown in Figure S4G, the mRNA levels of *Ece*, *Ace* and *Plg*, but not *Ide*, was also upregulated by Dio treatment in N2a cells, though the increase of these ADEs was not as high as NEP.

Several tryptophan metabolites, such as 5-hydroxyindole-acetic acid (5-HIAA) 16 and kynurenic acid 25, have been reported to regulate the expression and activity of NEP and decrease Aβ accumulation-induced brain toxicity by promoting Aβ degradation. Moreover, like the endogenous AhR agonists, they may also reduce the Aβ load in APP/PS1 mice and mild cognitive impairment patients through active AhR and alter the expression and activity of matrix metalloproteinases 47, 48. However, the relationship between AhR and NEP has not been clarified. Our study revealed that AhR could directly bound to the promoter region of *NEP* and modulated its transcription. As shown in Figure 4, two potential binding sites were identified in the *NEP* promoter. However, only a mutation in site1 reversed AhR-induced NEP expression. These results suggested a specific potential site for transcriptional regulation of the *NEP* gene by AhR. However, the other co-factors in the transcription complex and the possible epigenetic mechanism need to be further studied.

AhR plays an essential role in physiological and pathological processes in the CNS. Dysregulation of AhR is associated with aging and neurological disease. Ramos *et al.* 49 have reported that increased AhR expression was found in the cytosol of astrocytes in brain tissue of older adults and AD patients, apparently as microvesicles. The findings of Ramos *et al.* suggest that in older adults and AD patients, AhR's function as a transcription factor and its downstream signaling pathways is impaired. AhR also plays a critical role in regulating microglia activation and further affects astroglia function. Deletion of AhR in microglia resulted in a dysregulated proinflammatory transcriptional response 50. Interestingly, AhR is involved in both the proinflammatory and anti-inflammatory processes, depending on the AhR ligand present, e.g., the nature AhR ligand FICZ could inhibit LPS-induced inflammatory response in microglia 51. In the present study, we did not observe significant impacts on cognition in 8-month-old WT (Figure S3) or APP/PS1 mice (Figure 7) after AhR knockdown. This effect might due to the AAV9 mainly target to neurons rather than glial cells. Whether AhR knockdown in glial cells affects learning and memory ability, and whether AhR and its agonists affect the cognitive deficiency of APP/PS1 mice partially by regulating the inflammatory reaction caused by Aβ require further study.

Moreover, AhR expression in the intestine is regulated by a series of ligands, subsequently transferring signaling into the CNS through the gut-brain axis 52. Efficiently delivering therapeutics to the brain has long been a significant challenge in treating neurodegenerative diseases. Targeting the gut-brain axis may offer a comfortable and practical mode of drug administration. In the present study, we found that oral administration of diosmin effectively ameliorated the cognitive deficiency of APP/PS1 mice. Whether diosmin affects AhR in the intestine will be studied in the future.

As a ligand-activated transcription factor, AhR could recognize different ligands, including environmental, dietary, microbial, and metabolic cues, to control complex transcriptional programs 53. In the present study, we reported that several natural products, including diosmin and I3C, promoted Aβ clearance by increasing the activity of NEP. However, we should notice that NEP is also a therapeutic target of hypertension and heart failure 54. The agent for AD treatment through upregulates NEP should be safe and effective. Diosmin is a flavonoid isolated from citrus that is widely used in the clinical management of chronic venous insufficiency and varicose veins 55. Many studies showed that diosmin has various pharmacological activities, including anti-inflammation, anti-diabetes, anti-cancer and organ protection 55. I3C is a compound isolated from cruciferous vegetables and is used as a dietary supplement. Recent studies have shown that I3C has many biological effects on regulating autoimmunity and inflammation. I3C is considered to be a potential therapeutic agent for colitis 56, systemic lupus erythematosus 57, retinal degeneration 58, cerebral ischemic stroke 59, and Parkinson's disease 60. Herein, both diosmin and I3C showed a significant therapeutic effect on cognitive deficits through AhR activation and NEP upregulated. Our results suggest that nutritional therapy, including diet modification and the use of a nutritional supplement, is a promising approach for treating and/or preventing AD.

## Supplementary Material

Supplementary figures.

## Figures and Tables

**Table 1:** Nucleotide sequences of gene-specific primers used for quantitative real-time PCR

Gene name	Sequence of forward and reverse primers (5' to 3')
*Nep*	CCAGACTGATTCGTCAGGAAC
	TGACCTCCAGGGAAAAGTTGTT
*Ide*	CAGAAGGACCTCAAGAATGGGT
	GCCTCGTGGTCTCTCTTTATCT
*Ace*	AGGTTGGGCTACTCCAGGAC
	GGTGAGTTGTTGTCTGGCTTC
*Ece1*	TCTCCGAGGGCGATGTGTA
	CTTCTCCACCGAGGTCCGA
*Plg*	TGCAGTGGAGAAAAGTATGAGGG
	AGGGATGTATCCATGAGCATGT
*Cyp1a1*	GACCCTTACAAGTATTTGGTCGT
	GGTATCCAGAGCCAGTAACCT
*Cyp1b1*	CACCAGCCTTAGTGCAGACAG
	GAGGACCACGGTTTCCGTTG
*Gst*	CCTGGCTGCAGCAGGGGTGGAG
	CGGTTTTTGGTCCTGTCTTTTGC
*Gapdh*	TGTGTCCGTCGTGGATCTGA
	CCTGCTTCACCACCTTCTTGAT

## Abbreviations

AD: Alzheimer's disease
ADEs: amyloid degrading enzymes
AhR: aryl hydrocarbon receptor
Aβ: amyloid β
ChIP: chromatin immunoprecipitation assay
CNS: central nervous system
FBS: fetal bovine serum
I3C: indole-3-carbinol
IBD: inflammatory bowel disease
IDE: insulin-degrading enzymes
L-KN: L-Kynurenine
MS: multiple sclerosis
MWM: morris water maze
N2a: neuro-2a
NEP: neprilysin
PBS: phosphate-buffered saline
SR1: StemRegenin 1
WT: wild type
