# Supplementary material for: Activating AhR alleviates cognitive deficits of Alzheimer's disease model mice by upregulating endogenous Aβ catabolic enzyme Neprilysin
Source: Theranostics. 2021 Aug 11;11(18):8797–812. doi: 10.7150/thno.61601 (PMC8419060; doi:10.7150/thno.61601)
Supplement: Supplementary file 1 — Supplementary figures. [file thnov11p8797s1.pdf]

Supplementary material

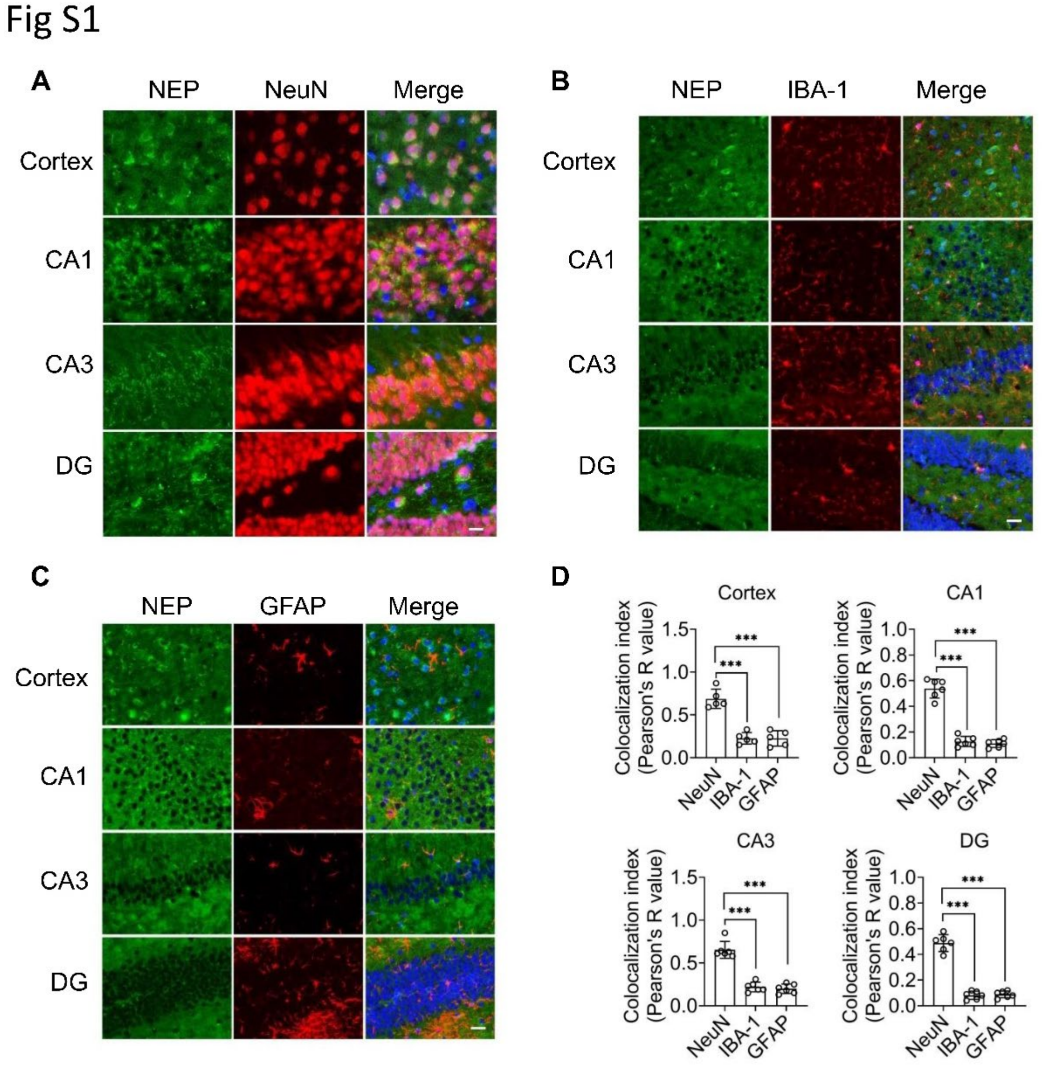

Figure S1. NEP staining in brain tissue

Brain tissues from APP/PS1 mice at 3-months-old were fixed and stained for NEP (green), IBA-1 (red) (A), GFPA (red) (B) and DAPI (blue). (D) The colocalization index of NEP and NeuN, IBA-1 or GFAP. n = 6. Each point represents the average of 3-5 sections values per mouse. Data were expressed as mean ± SD. *** $p < 0.001$ vs. the NeuN group. DG: dental gyrus; CA1 and CA3 of the hippocampus. Scale bar: 20 µm.

Fig S2

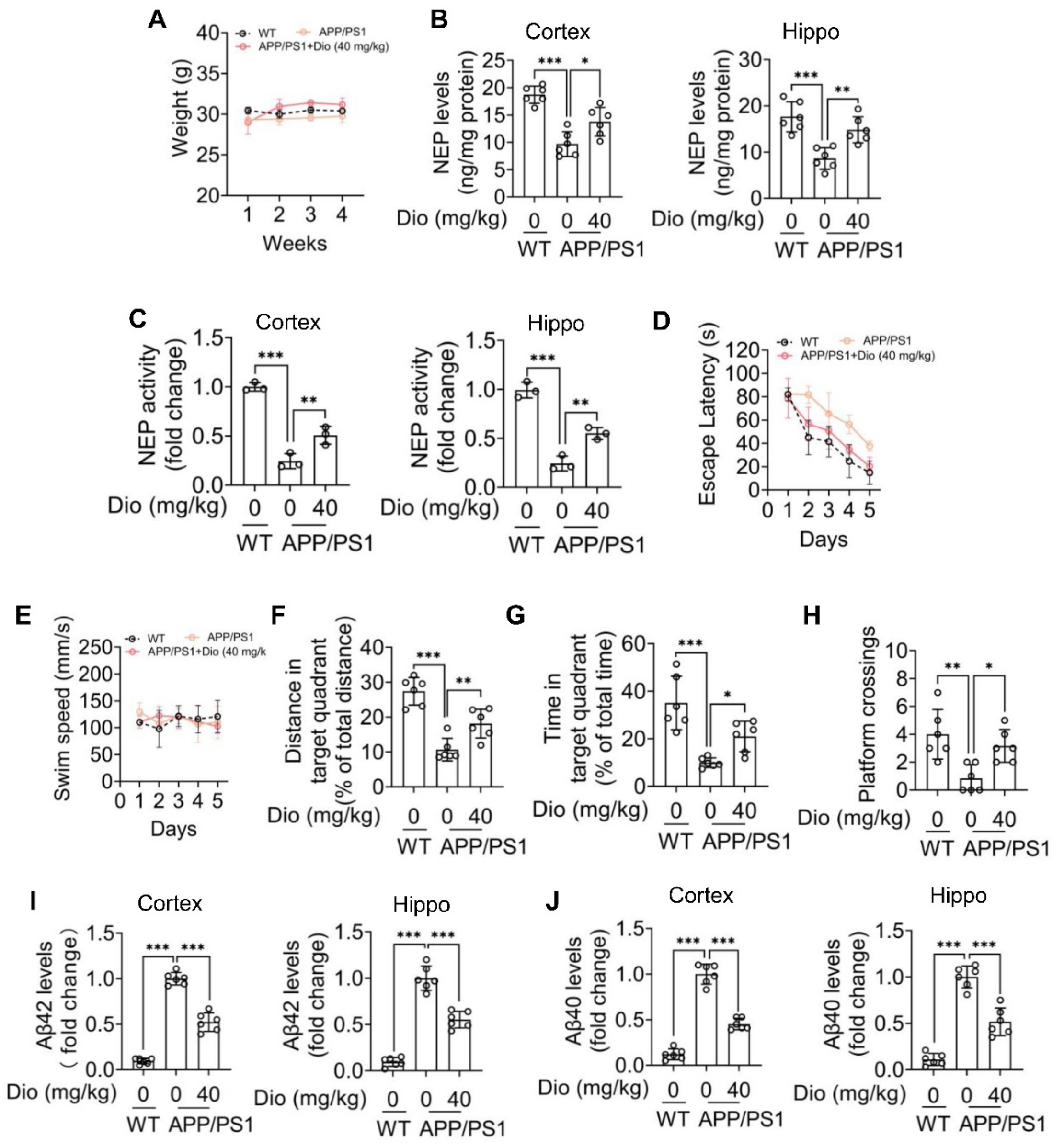

Figure S2. Diosmin increases functional NEP expression and ameliorates cognitive deficiency in female APP/PS1 mice

Female APP/PS1 mice (8 months of age) were administrated with vehicle or Dio (40 mg/kg) for 4 weeks. The WT littermates were used as control. After treatment, the mice were subjected to the MWM test. At the end of the treatment, the mice were sacrificed. The cortex and hippocampus tissues were collected and homogenized. The protein was extracted, NEP and soluble Aβ42 levels

were analyzed by ELISA. The NEP enzyme activity in the samples was detected by a NEP Activity Assay Kit. (A) The bodyweight was monitored during this period. n=6. (B) The NEP protein levels in the cortex and hippocampus. n = 6. (C) The NEP enzyme activity in the cortex and hippocampus. n=3. (D) Escape latency during spatial acquisition training. n=6. (E) Swim speed during the spatial probe test. (F) Distance in the target quadrant, (G) time spent in the target quadrant and (H) the number of platform crossings in the spatial probe test. n = 6. (I) The soluble Aβ42 levels and (J) soluble Aβ40 levels in cortex and hippocampus. n = 6. Data were expressed as mean ± SD. * $p < 0.05$, ** $p < 0.01$, *** $p < 0.001$ vs. indicated group. Dio: diosmin; Hippo: hippocampus; MWM: Morris water maze; WT: wildtype.

Fig S3

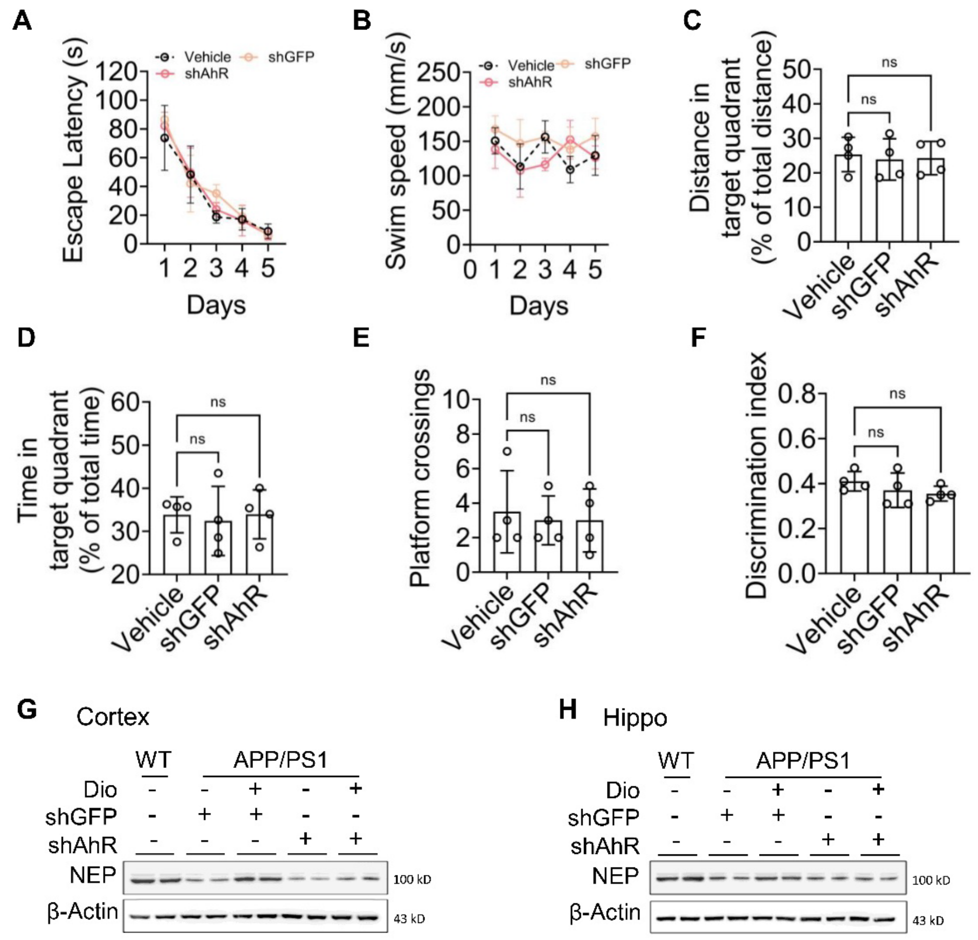

Figure S3. Effects of AhR knockdown through AAV9 injection on cognition ability of adult mice

The WT mice (8 months of age) were subjected to ICV injection with AAV9 carrying the shRNA targeting AhR (shAhR) or the shRNA targeting GFP (shGFP). Five days after injection, the mice were subjected to the MWM test. After the MWM test was completed, the mice were subjected to the object recognition test. (A) Escape latency during spatial acquisition training. (B) Swim speed during the spatial probe test. (C) Distance in the target quadrant, (D) time spent in the target quadrant and (E) the number of platform crossings in the spatial probe test. (F) The discrimination index of the object recognition test. n = 4. (G-H) APP/PS1 mice (8 months of age) received an

intracerebroventricular injection with AAV9 carrying the shRNA targeting AhR (shAhR) or the shRNA targeting GFP (shGFP). Five days after injection, the mice were administrated with vehicle or Dio (40 mg/kg) for another 4 weeks. Total protein from the cortex and hippocampus tissues was prepared to determine the NEP levels by western blotting. The original data of other animals that determine NEP levels through western blotting in Figure 3E were shown. Data were expressed as mean ± SD. MWM: Morris water maze. WT: wildtype.

Fig S4

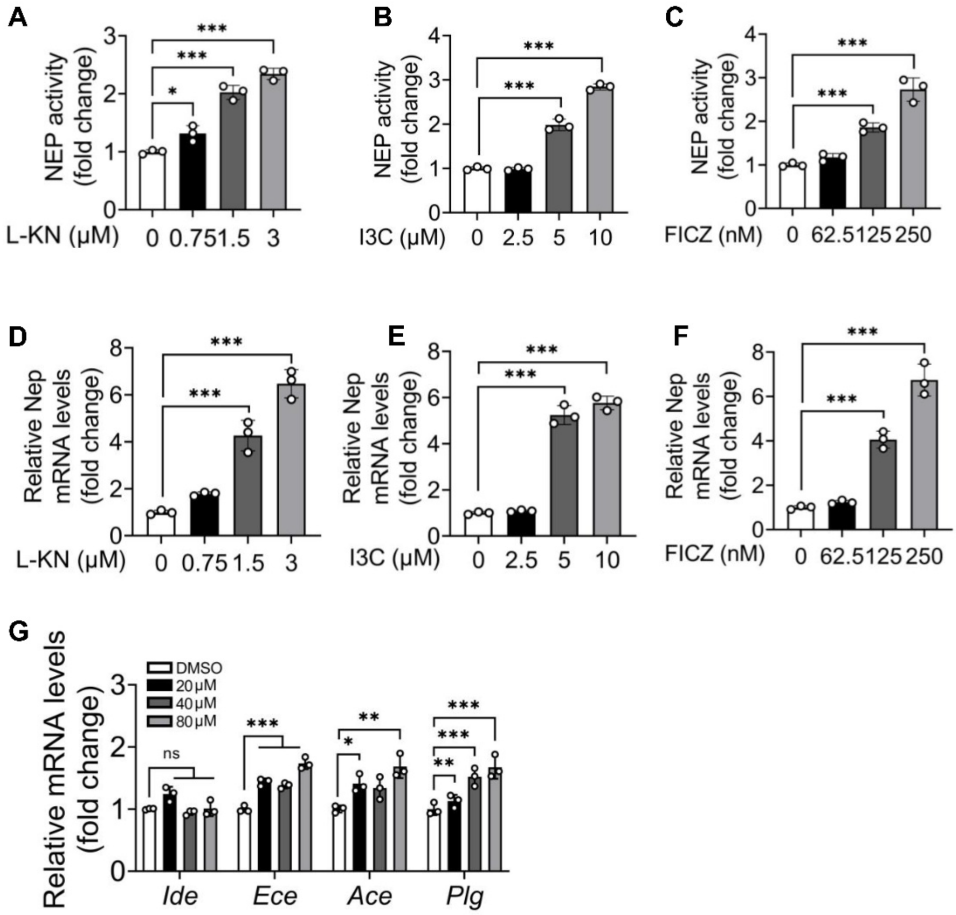

Figure S4. AhR agonists increase functional NEP expression and regulate other ADEs expression

N2a cells were treated with L-KN (A), I3C (B), and FICZ (C) at the indicated concentration for 12 h. The total protein were extracted and the NEP enzyme activity in the samples was detected by a NEP Activity Assay Kit. N2a cells were treated with L-KN (D), I3C (E), FICZ (F) or Dio (G) at the indicated concentration for 6 h, and then the total RNA was extracted. The mRNA levels of indicated genes were detected by qRT-PCR. n = 3, * $p < 0.05$, *** $p < 0.001$ vs. ctrl group (DMSO). Ace: angiotensin-converting enzyme; Ece: endothelin converting enzyme; Dio: diosmin; I3C: indole-3-carbinol; Ide: insulin degradation enzyme; L-KN: L-Kynurenine; NEP: neprilysin; Plg:

plasminogen.

Fig S5

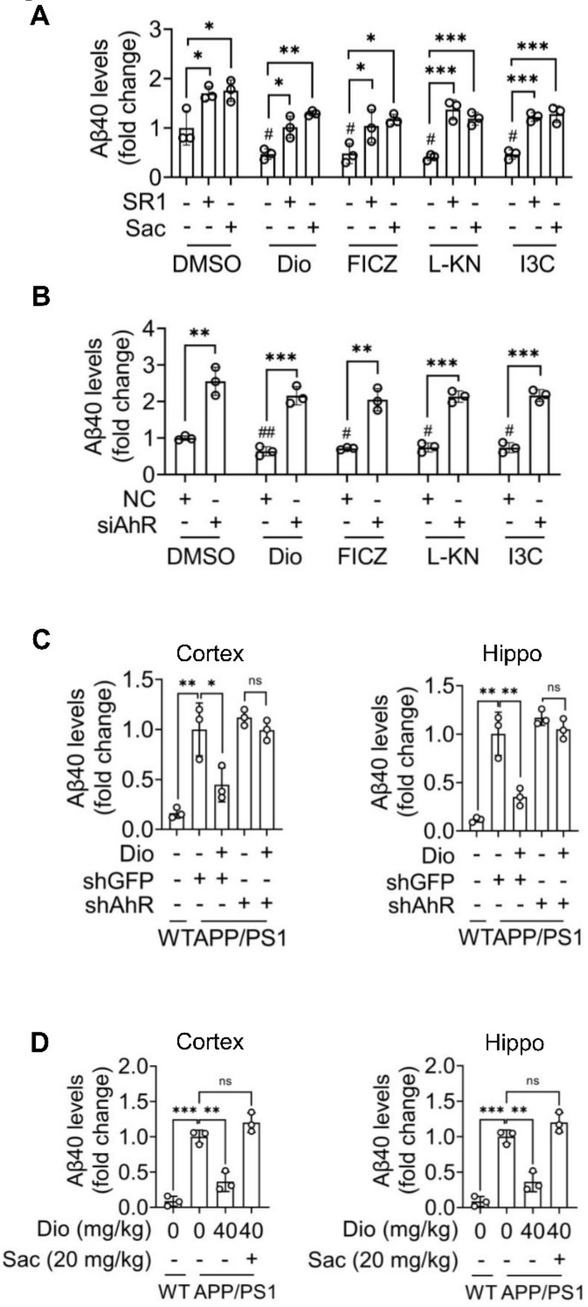

Figure S5. Activated AhR decreases Aβ40 levels in a NEP-dependent manner

(A) N2a-APP cells were treated with Dio (80 μM), FICZ (250 nM), L-KN (3 μM) or I3C (10 μM) with or without SR1 (2 μM) or Sac (1 μM) for 24 h, the supernatant was collected and the Aβ42 levels were determined by ELISA. (B) N2a-APP cells were transfected with non-targeting siRNA (NC) or siRNA targeting AhR (siAhR) for 48 h, and then administrated with the compounds

mentioned in (A) for an additional 24 h. The soluble Aβ40 levels in the supernatant were measured by ELISA. (C) APP/PS1 mice (8 months of age) were subjected to ICV injection with AAV9 carrying the shRNA targeting AhR (shAhR) or the shRNA targeting GFP (shGFP). Five days after injection, animals were administrated with vehicle or Dio (40 mg/kg) for another 4 weeks. (D) The APP/PS1 mice (8 months of age) were administrated vehicle or with Dio (40 mg/kg) with or without Sac (20 mg/kg) for 4 weeks. At the end of treatment, the mice were sacrificed. The cortex and hippocampus tissues were collected and homogenized. The soluble Aβ40 was extracted and analyzed by ELISA. n = 3. Data were expressed as mean ± SD. # $p < 0.05$, ## $p < 0.01$ vs. DMSO group; * $p < 0.05$, ** $p < 0.01$, *** $p < 0.001$ vs. the indicated group. Dio: diosmin; Hippo: hippocampus; I3C: indole-3-carbinol; L-KN: L-Kynurenine; Sac: Sacubitrilat; SR1: StemRegenin 1.
